# Effects of a brief mindfulness-meditation intervention on neural measures of response inhibition in cigarette smokers

**DOI:** 10.1371/journal.pone.0191661

**Published:** 2018-01-25

**Authors:** Catherine I. Andreu, Diego Cosmelli, Heleen A. Slagter, Ingmar H. A. Franken

**Affiliations:** 1 Escuela de Psicología, Pontificia Universidad Católica de Chile, Santiago, Chile; 2 Millennium Institute for Research in Depression and Personality (MIDAP), Santiago, Chile; 3 Centro Interdisciplinario de Neurociencias, Pontificia Universidad Católica de Chile, Santiago, Chile; 4 Department of Psychology, University of Amsterdam, Amsterdam, the Netherlands; 5 Amsterdam Brain and Cognition, University of Amsterdam, Amsterdam, the Netherlands; 6 Department of Psychology, Education & Child Studies, Erasmus University Rotterdam, Rotterdam, the Netherlands; Monash University, AUSTRALIA

## Abstract

Research suggests that mindfulness-practices may aid smoking cessation. Yet, the neural mechanisms underlying the effects of mindfulness-practices on smoking are unclear. Response inhibition is a main deficit in addiction, is associated with relapse, and could therefore be a candidate target for mindfulness-based practices. The current study hence investigated the effects of a brief mindfulness-practice on response inhibition in smokers using behavioral and electroencephalography (EEG) measures. Fifty participants (33 females, mean age 20 years old) underwent a protocol of cigarette exposure to induce craving (cue-exposure) and were then randomly assigned to a group receiving mindfulness-instructions or control-instructions (for 15 minutes approximately). Immediately after this, they performed a smoking Go/NoGo task, while their brain activity was recorded. At the behavioral level, no group differences were observed. However, EEG analyses revealed a decrease in P3 amplitude during NoGo vs. Go trials in the mindfulness versus control group. The lower P3 amplitude might indicate less-effortful response inhibition after the mindfulness-practice, and suggest that enhanced response inhibition underlies observed positive effects of mindfulness on smoking behavior.

## Introduction

Cigarette smoking is the largest preventable cause of death in the world and the costs associated with it correspond to more than $300 billion a year in the United States [1, 2]. Although smokers realize that smoking carries health risks, quitting smoking is notoriously hard and may require several attempts [3]. Some recent studies suggest that mindfulness-based practices are as or more effective in treatment for smoking cessation than conventional behavioral treatments such as Freedom from Smoking treatment, cognitive-behavioral approaches or 12-step programs [4–11].

Mindfulness practices involve attending to present-moment experiences, with non-judgmental and non-reactive awareness to internal and external stimuli [12]. Various studies have reported positive results for the efficacy of mindfulness programs in substance use and relapse prevention (Mindfulness-based relapse prevention, MBRP) [13–17], including studies using randomized-controlled trials with several weeks of mindfulness training [4, 9, 18, 19]. Reduced cigarette craving and withdrawal symptoms have also been observed after shorter mindfulness-based interventions [11, 20]. For example, Bowen and Marlatt used a brief 15 minute cue-exposure paradigm with mindfulness-based instruction and found a reduction in the number of cigarettes smoked 7 days after the intervention, as compared to a control group [10]. This finding suggests that mindfulness instruction can quickly alter smoking behavior.

Although there are theoretical proposals regarding the tentative psychological and neurobiological mechanisms underlying the effects of mindfulness training on addiction [21–24], few studies have so far empirically examined the neurocognitive effects of mindfulness-based practices on addiction [25, 26]. It has been hypothesized that mindfulness-based exercises modulate both bottom-up and top-down processes among individuals with substance use disorders [22]. Evidence for bottom-up modulation includes, for examples reduced stress reactivity after mindfulness training in alcohol and/or cocaine abusers [17] and decreased reactivity to smoking cues using a mindfulness-instruction in smokers as evidenced by decreased craving-related activity in the anterior cingulate cortex (ACC) [27]. There is also evidence for top-down changes, e.g., in inhibitory control, performance monitoring, and decision-making, related to mindfulness [28–34]. Notably, inhibitory control is disrupted in addictive behaviors, with several models of addiction highlighting its role in the development and maintenance of addiction [35–40]. This raises the possibility that mindfulness may affect addictive behaviors by improving response inhibition. Yet, how mindfulness may do so it still very much unclear. The present study aimed to investigate the neural mechanisms underlying the effect of a brief mindfulness-based practice on response inhibition in cigarette smokers.

In recent years, interest in mindfulness meditation as a practice to train cognitive functions has grown exponentially. Accumulating evidence suggests that meditation practice can modulate attention and executive functions [28, 32, 41–47] as well as emotional reactivity or regulation skills [41, 48–56]. Recent research also suggests that meditation may enhance cognitive control. Experienced meditators and novices, who had 16 weeks of mindfulness training, displayed better performance on Stroop interference tasks [29, 45, 47] and response inhibition tasks [28, 57]. Improvements in conflict monitoring using the Attention Network Test (ANT) [58] have also been found in experienced meditators [59] after a week-long meditation retreat [60] and after just 5 days of meditation training in novices [30]. Functional magnetic resonance imaging (fMRI) studies have shown that ACC and prefrontal activity is modulated by meditation, with experienced meditators exhibiting increased activation and stronger connectivity in and between these areas [61–64].

Although these studies are indicative of enhanced cognitive control in meditators, effects of meditation on neurobiological measures of response inhibition have not been studied yet in the context of addiction. Given that response inhibition is a core function impaired in addictions and a main mechanism through which mindfulness practices may act to decrease smoking addiction or prevent relapse, it is essential to determine if mindfulness-based practices can indeed modulate behavioral and/or neural indices of response inhibition.

Response inhibition has been measured using Go/NoGo tasks with behavioral measures (omission errors in Go trials; commission errors in NoGo trials; reaction times, RTs) [65]. Two event-related potentials (ERPs) have been used as neural indices of response inhibition, the N2 and P3, as both are associated with larger amplitudes in NoGo trials compared to Go trials and thus reflect changes in brain activity needed to inhibit responses in a Go/NoGo task [65]. The N2 is a negative wave emerging 200–300 ms after stimulation and is thought to represent top-down mechanisms necessary to inhibit an automatic response and detect conflict during early stages of inhibition [65–68]. On the other hand, the P3 is a positive wave that emerges 300–500 ms after stimulus onset and is thought to reflect a later stage of the inhibitory process, related to the inhibition of the motor system itself [65, 69–72]. Deficits in response inhibition indices (behavioral or neural) have been described in several substance-dependent patient populations, including cigarette smokers [39, 40]. The specific effect on response inhibition varies between different types of addictions, but the most consistent finding is lower N2 amplitudes in addicted populations relative to controls, accompanied by reduced task performance or increased error rates in a Go/NoGo task [39]. In a clear example, Luijten et al. found reduced N2 amplitudes and diminished accuracy on a smoking Go/NoGo task in smokers compared to controls [40].

To test the effects of a mindfulness-based practice on response inhibition in an addiction context, using EEG, we investigated the effects of a previously developed brief mindfulness intervention [10] on behavioral and neural responses on a Go/NoGo task in cigarette smokers [40]. We expected augmented response inhibition indices in smokers after the brief mindfulness-based intervention as compared to smokers after a control intervention. More specifically, we expected lower error rates (particularly commission errors) in the mindfulness compared to control group, accompanied with increased N2/P3 amplitudes. In addition, we expected these behavioral and/or electrophysiological effects to be particularly pronounced in trials with smoking-related compared to neutral pictures. Finally, we expected higher post-intervention craving in the control group than in the mindfulness group.

## Methods

### Participants

Fifty cigarette-smokers participated in this study. Subjects had to be 18 years or older, smoke daily, be interested in cutting down or quitting smoking, and not be currently enrolled in a treatment or program. Exclusion criteria included current abuse of a substance other than nicotine, a current diagnosis of a physical or psychiatric illness and previous experience with mindfulness meditation. Eligible participants were randomly assigned to the mindfulness or control group. 25 smokers in the mindfulness group (17 females, Mean Age [MA] = 20.00, Standard Deviation [SD] = 1.72) and 25 smokers in the control group (16 females, MA = 20.6, SD = 1.75) completed the experiment. The mean age (*t* (48) = -1.2, *p* = 0.23) and gender ratio (chi-square = 0.09, *p* = 0.76) of both groups did not differ. Participants were undergraduate students, who received course credits for their participation. The local ethics committee approved the protocol of the study (number 2015–7) and participants provided written informed consent.

### Procedure

**Fig 1:**
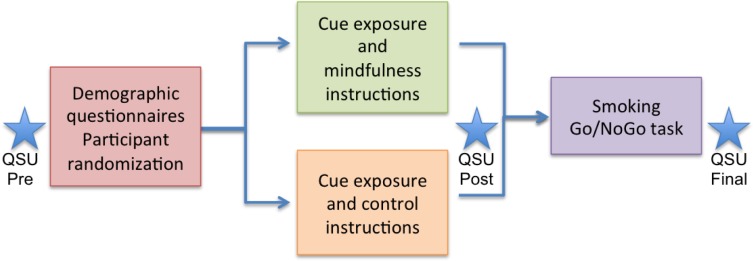
Scheme of the experimental procedure. Questionnaire application, group randomization and task performance are shown. Blue stars represent the Questionnaire of Smoking Urges (QSU) application at three different time points (pre, post and final).

Participants were instructed to abstain from smoking for at least two hours before the experiment to reduce the acute effects of nicotine on ERP amplitudes [73]. After arrival, participants approved participation by signing an informed consent and completed the questionnaires. Participants were then seated in a light and sound-attenuated room. Electrodes were applied and the experiment was explained. Following a previous study [10], participants then completed a cue-exposure protocol and received either mindfulness-based or control coping instructions via audio recordings. As in the previous study, the cue-exposure protocol was delivered in four stages: open a pack of cigarettes, place a cigarette on the table in front of you, place the cigarette in your mouth and, finally, bring a lighter to the cigarette without igniting the cigarette. These instructions were the same for the mindfulness and control groups. Instructions to cope with cue-induced smoking-related thoughts or cravings that arose were different, however. Control participants were asked to use any techniques that they would naturally use to cope with urges or one they had used in the past. At the end of the cue-exposure protocol, control participants reported that they used distraction techniques to cope with smoking urges, allowing us to verify that they did not employ a mindfulness-related technique. The mindfulness group received instructions to accept feelings, sensations, or thoughts in a mindful, nonjudgmental way. They were also given instructions for “urge-surfing”, a technique often included in MBRP treatment for substance use [8, 10]. The duration of the audio sets was the same for both groups, approximately 15 minutes. Then, a modified smoking Go/NoGo task was performed while EEG and behavioral data were collected (see Fig 1 for a schematic illustration of the procedure). Additionally, participants performed an Eriksen-Flanker task after the Go/NoGo task (results not reported here).

### Instruments

All participants completed a questionnaire concerning demographic data (i.e. age, gender, level of education), the amount of cigarettes smoked per day and the Fagerstrom Test for Nicotine Dependence (FTND) [74, 75]. Participants also completed the brief version of the Questionnaire of Smoking Urges (QSU-brief) [76, 77] to assess subjective craving for a cigarette at three different time points of the experiment: before and after the intervention (pre, post) and at the end of the experiment (final).

### Smoking Go/NoGo task

A previously developed smoking-related Go/NoGo task [40] was modified for this study. A series of smoking or neutral pictures were presented. Each picture was displayed for 200 ms and had a blue or yellow frame. Frame color indicated whether a stimulus was a Go or NoGo trial. Each stimulus was followed by a black screen for a randomly varying duration between 1000 ms and 1500 ms. Participants were instructed to respond to the pictures in Go trials by pressing a button with the right index finger as fast as possible, and to withhold their response in NoGo trials. About 25% of all trials were NoGo trials and the proportion of smoking and non-smoking pictures in the task was equal. At most, four Go and two NoGo trials were presented consecutively. Participants completed the task in two blocks of 240 trials each, one with smoking pictures and one with neutral pictures. Block order was randomized and in the middle of the task an additional block with 18 emotionally positive pictures was used to washout possible carry-over effects (EmoMadrid affective picture database, http://www.uam.es/CEACO/EmoMadrid.htm) [78], but EEG was not analyzed during the additional block. Participants were given the opportunity to take a short break four times during the task. Before the start of the task, participants performed 23 practice trials, involving additional neutral pictures. Total task duration was about 15 minutes.

### EEG recording and processing

The EEG was recorded using Biosemi Active-Two amplifier system (Biosemi, Amsterdam, the Netherlands) from 33 scalp sites (following the 10–20 International system with one additional electrode at FCz) with Ag/AgCl electrodes mounted in an elastic cap. Six additional electrodes were attached to left and right mastoids, the two outer canthi of both eyes (HEOG), and infraorbital and supraorbital regions of the eye (VEOG). BrainVision Analyzer2 (Brain products GmbH, Munich, Germany) was used to process the data offline. All signals were digitized with a sampling rate of 512 Hz and a 24-bit A/D conversion with a bandpass of 0–134 Hz. Data were referenced offline to average mastoids. Offline, EEG and EOG activity was bandpass filtered between 0.1–30 Hz (phase shift-free Butterworth filter; 24 dB/octave slope). Data were next segmented in epochs of 1 s (200 ms before and 800 ms after stimulus onset). Ocular correction was applied using the Gratton and Coles algorithm [79], and epochs with an EEG signal exceeding ±100 mV were excluded from the average. The mean 200 ms pre-stimulus period served as a baseline. After baseline correction, average ERP waves were calculated for artifact-free trials at each scalp site separately for the four conditions of the smoking Go/NoGo task (all correct Go neutral, Go smoking, NoGo neutral, NoGo smoking trials). The N2 was defined as the mean value in the 200–300 ms time interval after stimulus onset, and was studied at a cluster of frontocentral electrodes, including Fz, FC1, FC2, FCz and Cz [40]. The P3 was defined as the mean value in the 300–450 ms time window after stimulus onset, and was studied at a cluster of central electrodes including FCz, Cz, C3, C4 [40]. One participant of the mindfulness group was excluded from the N2/P3 analyses because less than 20 artifact-free ERP epochs in at least one of the conditions remained after preprocessing, which is considered too few to obtain a reliable N2 and P3 [80]. The mean number of analyzable Go and NoGo epochs for the N2 and P3 components was 161.2 (SD = 26.07) and 43.9 (SD = 7.55) for neutral pictures and 161.9 (SD = 20.09) and 42.1 (SD = 6.92) for smoking pictures, respectively. The mean number of available error-epochs did not differ between groups for Go trials, *t* (47) = -0.51, *p* = 0.61 and for NoGo trials, *t* (47) = -0.64, *p* = 0.52.

### Statistical analysis

Repeated Measure ANOVAs (RM-ANOVAs) (with Greenhouse–Geisser adjusted p-values when appropriate) were used to analyze error rates and reaction time data of the Go/NoGo task, as well as the ERPs. The between-subjects factor in all RM-ANOVAs was Group (Mindfulness versus Control). The RM-ANOVAs examining behavioral effects, two within-subject factors were included: Inhibition (Go versus NoGo) and Picture (smoking versus neutral). The RM-ANOVAs examining ERP effects included a third within-subject factor Electrodes (Fz, FC1, FC2, FCz and Cz for N2; FCz, Cz, C3 and C4 for P3). Additionally, a 2x3 (Group x Time) RM-ANOVA was performed to analyze changes in the QSU-brief in time, and a t-test to compare the final-pre difference score in the QSU-brief between groups.

## Results

### Questionnaires

**Fig 2:**
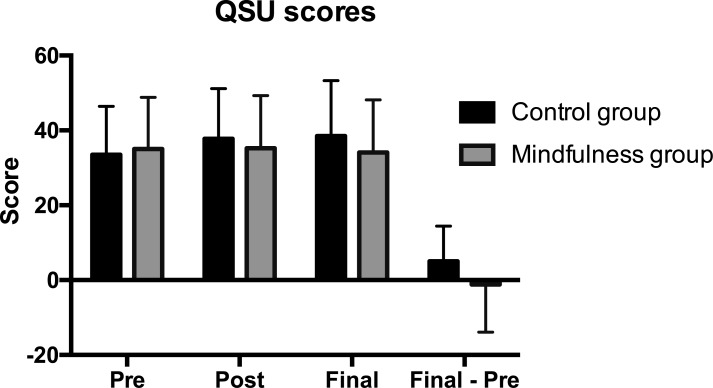
Smoking urge scores. This figure displays smoking urge scores for the control group (black) and mindfulness group (gray) separately for the pre, post and final time measurements, as well as the final minus pre (final–pre) difference. The control group shows increased smoking urges over time, contrary to the mindfulness group.

The number of daily cigarettes, the FTND score and the QSU-brief score per time point are presented in Table 1. There was no difference between groups in the number of daily smoked cigarettes, *t*(48) = 0.914, *p* = 0.37. Also, there was no difference between groups on the level of nicotine dependence measured by the FTND, *t*(48) = 0.4, *p* = 0.69, where both groups showed low levels of dependence. There was no significant change in the QSU-brief score measured by time, as no main group or time effect, nor group x time interaction was found (all *p* values > 0.11). Interestingly, a trend towards significance was found when the difference of the final QSU score minus pre QSU score was post hoc compared between groups, *t*(47) = -1.948, *p* = 0.057, with an increase in the QSU-brief score only in the control group, suggestive of increased smoking-urges in the control, but not the mindfulness group (Table 1). Fig 2 shows the QSU results for control and mindfulness groups for the three measured times and the final minus the pre score.

**Table 1:** Instrument scores and behavioral data for both groups.

	Mindfulness group	Control group	Test statistic	*p*
Age	20.0 (1.72)	20.6 (1.75)	*t* = -1.2	0.23
Gender, *n* (% female)	17 (68)	16 (64)	χ^2^ = 0.09	0.76
Cigarettes	9.0 (3.8)	7.9 (4.5)	*t* = 0.91	0.37
FTND	2.3 (1.5)	2.1 (1.9)	*t* = 0.4	0.69
QSU pre	35.0 (13.8)	33.5 (12.9)	*F* = 0.26	0.61
QSU post	35.2 (14.1)	37.8 (13.4)		
QSU final	34.1 (14.1)	38.5 (14.8)		
QSU final-pre	-1.17 (12.7)	5.04 (9.4)	*t* = -1.94	0.057
Go error neutral	0.02 (0.04)	0.01 (0.03)	*F* = 1.63	0.21
Go error smoking	0.02 (0.02)	0.02 (0.02)		
NoGo error neutral	0.21 (0.1)	0.15 (0.1)		
NoGo error smoking	0.21 (0.1)	0.2 (0.1)		
RT Go neutral	315.2 (46.7)	316.1 (57.8)	*F* = 0.09	0.75
RT Go smoking	316.9 (56.9)	306.8 (53.7)		

Demographic data, number of daily cigarettes, Fagerstrom Test for Nicotine Dependence (FTND) and questionnaire of smoking urges (QSU-brief) scores per time point (pre, post and final) are shown for both groups. Also, error rates (for Go and NoGo trials) and reaction times (RT, in milliseconds) on the smoking Go/NoGo task are shown for both groups separately. Data shown represent means, and standard deviations are in parentheses. Pre and post time points indicate before and after intervention and final time point is at the end of the complete experiment. Statistics for group analyses are shown.

### Behavioral data

Error rates and reaction times on the smoking Go/NoGo task are presented in Table 1. As expected, participants generally made more errors in NoGo trials compared to Go trials, as indicated by a significant main inhibition effect, *F*(1,48) = 221.3, *p*<0.001. Yet, contrary to our prediction, the brief mindfulness intervention was not associated with improved performance on the task, as there was no difference in error rate between groups, *F*(1,48) = 1.635, *p* = 0.21. Additionally, there was no difference between groups in error rates between Go and NoGo trials, as no Group x Inhibition interaction was found, *F*(1,48) = 1.15, *p* = 0.29. No main or interaction effects for Picture were found for error rates. Regarding reaction times in Go trials, both groups responded equally fast, as no main Group effect was found (*F*(1,48) = 0.098, *p* = 0.75). Also, there was no significant main effect for Picture, nor a Group x Picture interaction (all ps > 0.15).

### Effects on ERP

#### N2

**Fig 3:**
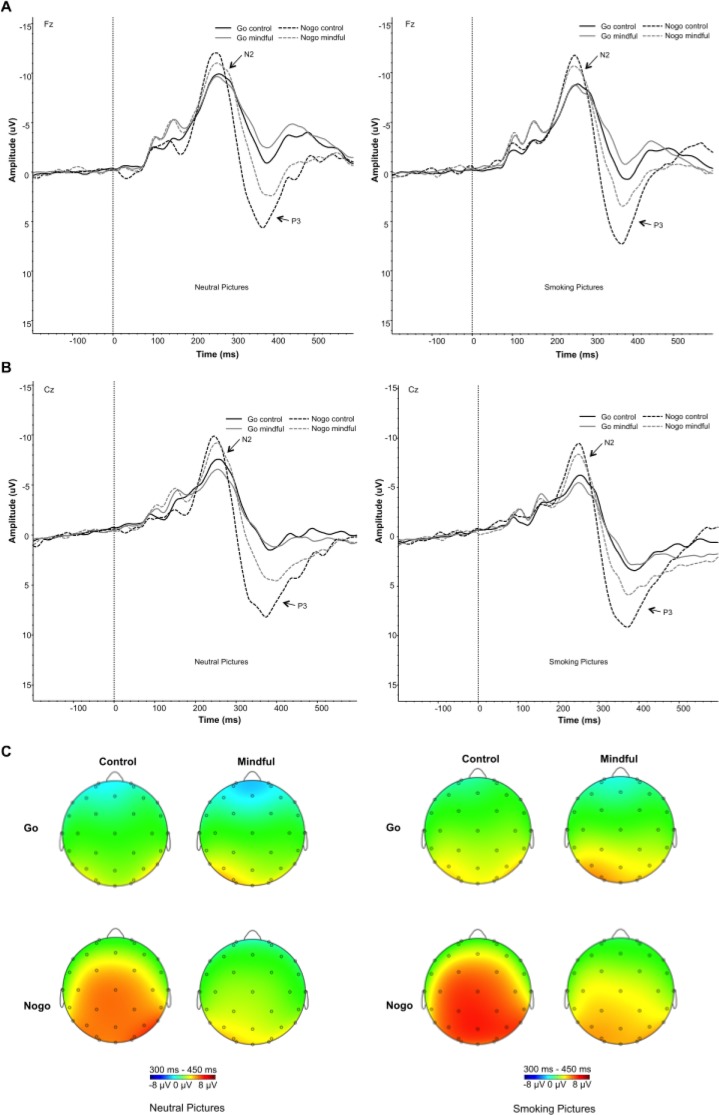
Effects of mindfulness on neural indices of response inhibition. Shown are grand-average stimulus-locked ERP waveforms for neutral (left) and smoking pictures (right) at Fz (Panel A) and Cz (Panel B), separately for correct Go and NoGo trials and the mindfulness and control group. Scalp voltage maps are shown in Panel C for mean amplitude for 300–450 ms. As can be seen, the mindfulness group displayed a reduced NoGo P3 compared to the control group.

Fig 3 shows the grand average ERP waveforms for neutral and smoking-related pictures separately for both groups at electrodes Fz and Cz. As expected, the N2 amplitude in NoGo trials was larger than in Go trials, as evidenced by a robust main effect of Inhibition, *F*(1, 47) = 13.18, *p* = 0.001. Yet, the two groups did not differ in N2 amplitude, as reflected in non-significant main Group and Group x Inhibition interaction effects (all *p values* > 0.6). N2 amplitudes were larger for neutral pictures than for smoking-related pictures, with a main Picture effect, *F*(1,47) = 7.28, *p* = 0.01. This N2 amplitude difference was not modulated by group or trial type (NoGo vs Go), as no significant Group x Picture interaction, nor Picture x Inhibition interaction was observed (all *p values* > 0.3). The main effect of Electrode was significant, *F*(4,188) = 48.91, *p*<0.001, reflecting a larger N2 at Fz. The Electrode x Inhibition interaction effect was significant as well, *F*(4,188) = 4.39, *p* = 0.013.

#### P3

As to the P3, as expected, there was a main effect of Inhibition on P3 amplitude (*F*(1, 47) = 44.84, *p*<0.001), with larger P3 amplitudes during NoGo trials than during Go trials. Notably, this inhibition effect was different between groups, as expressed by a Group x Inhibition effect, *F*(1,47) = 5.34, *p* = 0.025, reflecting smaller NoGo P3 amplitudes in the mindfulness group (Fig 3). The main Group effect was not significant, *F*(1,47) = 1.05, *p* = 0.3. P3 amplitudes were larger for smoking pictures than for neutral pictures, with a main Picture effect, *F*(1,47) = 21.16, *p*<0.001. There was no difference between groups in the smoking-related P3 effect, as no significant Group x Picture interaction, nor Group x Picture x Inhibition interaction was observed, nor did response inhibition modulate this effect, as reflected by a non-significant Picture x Inhibition interaction, (all *p values* > 0.5). The main effect of Electrode was significant, *F*(3,141) = 10.33, *p*<0.001, indicative of a maximal P3 at electrodes C3 and Cz. The Electrode x Inhibition interaction was also significant, *F*(3,141) = 57.2, *p*<0.001.

## Discussion

In this EEG study, we used a previously-developed brief mindfulness-based protocol for cigarette smoking [10] to examine effects of mindfulness on (neural makers of) response inhibition, an important cognitive control process hypothesized to be targeted by such practices. To this end, participants performed a smoking Go/NoGo paradigm [40] while we recorded their brain activity after a mindfulness-based or a control intervention. Contrary to our prediction, the brief mindfulness intervention was not associated with changes in behavioral response inhibition. Yet, notably, at the neural level, compared to the control group, the mindfulness group showed a reduced NoGo P3, though task performance was the same between groups. Although the explanation of this finding is not straightforward, the lower NoGo P3 in the mindfulness group may reflect reduced effort to reach a similar level of performance as the control participants [78, 81–83]. Together, these results may suggest that response inhibition is an important neurobiological mechanism targeted by mindfulness-based practices to cope with cigarette smoking.

The absence of behavioral improvements in response inhibition may be explained by the short duration of the mindfulness intervention or other factors, such as that the task used may not be sensitive to changes by brief mindfulness interventions. Fifteen minutes may not be long enough to behaviorally influence response inhibition measures in naïve participants using a Go/NoGo task. Indeed, previous studies using brief mindfulness practices (around 15 minutes) did not observe behavioral differences using a Flanker task [33]. In contrast, Bowen and Marlatt using a similar 15 minute cue-exposure paradigm with mindfulness-based instructions reported a reduction in the number of cigarettes smoked 7 days after the intervention, as compared to a control intervention [10]. As we did not include a follow-up measure, it is unclear if the mindfulness intervention may have also been associated with delayed positive effects in the current study. Also, the Go/NoGo task assesses inhibition of habitual responses, which may not be modifiable by short interventions. Other measures of inhibition such as the Stroop task may be more sensitive to changes in inhibitory control induced by short interventions. Future studies are necessary to establish this. Indeed, several studies have found effects of longer mindfulness interventions on Stroop measures [45, 47, 62]. Other studies have shown that mindfulness interventions may be effective in reducing executive deficits in substance abusers [84] and may enhance brain functions associated with self-control capacities in smokers [26]. Interestingly, using a brief mindfulness meditation may be an efficient strategy to foster self-control under conditions of low resources [85]. However, more studies evaluating the effects of brief mindfulness interventions and the mechanisms underlying effects are certainly needed.

In our study, there was no effect of picture content (smoking vs. neutral) on task performance. Luijten et al. previously also did not find an effect of picture content on performance accuracy in a similar smoking Go/NoGo task [40]. The relatively low level of cigarette dependence of the participants in the current study could explain the absence of an effect of smoking pictures on response inhibition. Interestingly, a trend for a difference between groups was observed in the final-before difference score, reflecting a selective increase in self-reported craving only for the control group (Table 1). Albeit speculative, this may suggest (but note, only trend level) that the mindfulness group did not display the expected increase in craving. A recent meta-analysis of randomized controlled trials of mindfulness treatments for substance misuse revealed significant moderate to large effects of mindfulness treatments on craving [86]. Hence, it should be studied further whether a brief mindfulness intervention can also reduce craving in smokers.

Task performance is of prime importance in the interpretation of neural activation measured by fMRI or EEG. Some studies have found an increase in the activation of response-inhibition related areas or in the amplitude of NoGo P3 in clinical populations, such as children with attention-deficit/hyperactivity disorder or substance abusers [39, 78, 81, 83], while showing performance comparable to that of a control group. This result is often interpreted as more effortful or a less efficient response inhibition. Notably, in this study, we obtained the opposite result, namely equal task performance accompanied by a decrease in the amplitude of NoGo P3 in the mindfulness group. Albeit speculative, this could be interpreted as less effortful response inhibition in smokers who participated in the mindfulness exercise.

Interestingly, mindfulness only modulated the P3, but not the N2 component, suggesting that the effect of this brief mindfulness-based practice may be limited to the actual inhibitory process. Using a Stroop task, increased parietal N2 amplitudes have been described in older adults after an 8-week mindfulness intervention [87] and in naïve participants after a 16-week mindfulness intervention [45]. In light of our results, it may be possible that longer interventions are needed to modulate processes reflected in the N2 component. Of further note, Moore et al. also found a decrease in P3 amplitude after the 16-week mindfulness intervention [45]. A decreased P3 amplitude is also in line with previous studies showing that meditation practice leads to more effective brain resource allocation [46] and improved efficiency (reduced fMRI activation) in sustained attention and impulse-control related brain areas [88], as well as a reduced Pe amplitude—a P3-like component related to error awareness [33, 89]. Yet, the effect of the mindfulness practice on P3 amplitude was observed regardless of the nature of the picture (smoking or neutral), indicating that mindfulness may have modulated response inhibition more generally.

One limitation of this study is that we did not measure changes in mindfulness states during the experiment, rendering it unclear if the intervention specifically modified the mindfulness state during the experiment. Additionally, because of the present study design without a baseline measure, we cannot discard pre-existing differences between groups. Participants were randomized into one of the two groups and recent guidelines argue that in this case, there is no need for baseline difference testing [90]. Moreover, it must be kept in mind that all smokers in this study were young smokers with a relatively low level of dependence, so generalization to other categories of smokers is limited. Their low level of dependence may have also prevented us from observing stronger effects of mindfulness on smoking-related processes. Additionally, we used a brief mindfulness-meditation, and future studies using longer mindfulness interventions, such as the 8-week MBRP program, are required to determine longer-term effects of mindfulness on smoking behavior.
